# Dietary Supplementation of *Calendula officinalis* Counteracts the Oxidative Stress and Liver Damage Resulted from Aflatoxin

**DOI:** 10.5402/2013/538427

**Published:** 2013-02-12

**Authors:** Mohamed A. Hamzawy, Ezzeldein S. M. El-Denshary, Nabila S. Hassan, Fathia A. Mannaa, Mosaad A. Abdel-Wahhab

**Affiliations:** ^1^Pharmacology and Toxicology Department, College of Pharmacy, Misr University for Science and Technology, Al-Motamayez District, P.O. Box 77, 6th October City, Egypt; ^2^Pharmacology and Toxicology Department, Faculty of Pharmacy, Cairo University, Cairo 11787, Egypt; ^3^Pathology Department, National Research Center, Dokki, Cairo 12311, Egypt; ^4^Medical Physiology Department, National Research Center, Dokki, Cairo 12311, Egypt; ^5^Food Toxicology and Contaminants Department, National Research Center, Dokki, Cairo 12311, Egypt

## Abstract

This study was conducted to evaluate the total phenolic compounds, the antioxidant properties, and the hepatorenoprotective potential of *Calendula officinalis* extract against aflatoxins (AFs-) induced liver damage. Six groups of male Sprague-Dawley rats were treated for 6 weeks included the control; the group fed AFs-contaminated diet (2.5 mg/kg diet); the groups treated orally with *Calendula* extract at low (CA1) and high (CA2) doses (500 and 1000 mg/kg b.w); the groups treated orally with CA1 and CA2 one week before and during AFs treatment for other five weeks. The results showed that the ethanol extract contained higher phenolic compounds and posses higher 1,1-diphenyl 1-2-picryl hydrazyl (DPPH) radical scavenging activity than the aqueous extract. Animals fed AFs-contaminated diet showed significant disturbances in serum biochemical parameters, inflammatory cytokines, and the histological and histochemical pictures of the liver accompanied by a significant increase in malondialdehyde (MDA) and a significant decrease in superoxide dismutase (SOD) and glutathione peroxidase (GPx) in liver. *Calendula* extract succeeded to improve the biochemical parameters, inflammatory cytokines, decreased the oxidative stress, and improved the histological pictures in the liver of rats fed AFs-contaminated diet in a dose-dependent manner. It could be concluded that *Calendula* extract has potential hepatoprotective effects against AFs due to its antioxidant properties and radical scavenging activity.

## 1. Introduction

Mycotoxins are fungal metabolites toxic to humans and animals, commonly found as contaminants of food or feed [1]. Aflatoxins (AFs) are principally produced by *Aspergillus flavus *and *Aspergillus parasiticus*. Among these, aflatoxin B_1_ (AFB_1_) is the predominant form as cereal and oilseed contaminants and presents the highest toxic potential [1], being hepatotoxic and carcinogenic in human and animals [2]. The biotransformation of AFB_1_ by liver microsomal enzymes resulted in toxic metabolites AFB_1_-8, 9-epoxide. The toxic effects of AFs mostly arise from the binding of this particular epoxide derivative to cellular macromolecules such as DNA and proteins [3]. AFB_1_ is classified by the International Agency of Research on Cancer (IARC) as Group 1 carcinogen [4]. This mycotoxin is also mutagenic, teratogenic, and immunosuppressive in farm and laboratory animals [5] and mainly affects the cell-mediated immunity [6]. Reactive oxygen species such as the hydroxyl radical, superoxide anion, and hydrogen peroxide, which are generated as a result of this metabolism, are also involved in the toxic mechanism of AFs [7–10].

*Calendula officinalis* L., a member of the Asteraceae family, is an annual plant with yellow to orange flowers, mostly seen in the Mediterranean region and has been cultivated as a food and medicinal plant since the Middle Ages [11]. It has been used in the treatment of inflammation and skin wounds [12]. In the early Indian and Arabic cultures, as well as in ancient Greece and Rome, *C. officinalis* was used as colourant for fabrics, foods, and cosmetics [13]. Nowadays, *C. officinalis* is approved for food use in USA and appears in the Food and Drug Administration's list of GRAS (Generally Recognized as Safe) substances. It has a high economic value as an herbal medicine and is widely used in cosmetics, perfumes, pharmaceutical preparations and in food [11]. *C. officinalis* contains a high number of carotenoids such as flavoxanthin, lutein, rubixanthin, *β*-carotene, g-carotene, and lycopene [14]. It has been cited that *β*-carotene may work synergistically with vitamin E [15]. Lycopene, another carotenoid, has been found to have antioxidant, antimicrobial, and antiproliferative properties. Research suggests that it can be very protective against prostate cancer [16]. *C. officinalis* has been studied extensively for its beneficial effects on humans. Literature has shown that an herbal tea made from *C. officinalis* could improve the symptoms of colitis, duodenal ulcers, and gastroduodenitis [16]. Although considerable work has been done on *C. officinalis* extracts, no report is available on its role against the oxidative stress generated by natural toxicants. Therefore, the aims of the present study was to determine the total phenolic content, the radical scavenging activity of the ethanolic and aqueous extract of *Calendula in vitro* and to evaluate the possible hepatoprotective effects of the extract against oxidative stress induced by aflatoxins in rats.

## 2. Material and Methods

### 2.1. Chemicals and Kits

Aflatoxins standards were purchased from Sigma Chemical Co., (St. Louis, MO, USA). Alanine aminotransferase (ALT), aspartate aminotransferase (AST), glutathione peroxidase (GPx), and superoxide dismutase (SOD) were purchased from Randox (Antrim, UK). Alkaline phosphatase (ALP), total protein (TP), albumin, and creatinine were purchased from QCA (AMPOSTA, Spain). Urea was purchased from Prodia (Korbach, Germany). Lipid peroxide formation was evaluated as malondialdehyde (MDA) and was purchased from Oxis Research Co. (USA). Alpha fetoprotein (AFP) was purchased from Monobind Inc. (Lake Forest, USA). Interleukin-1*β* (IL-1*β*) and tumor necrosis factor-alpha (TNF-alpha) were purchased from Orgenium (Helsinki, Finland). All other chemicals were of the highest analytical grade available.

### 2.2. Aflatoxin Preparation

Aflatoxins were produced via fermentation of rice by *Aspergillus parasiticus* NRRL 2999. The fermented rice was autoclaved, dried, and ground to a powder, and AFs content was measured by HPLC [17]. The rice powder was incorporated into the basal diet to provide the desired level of 2.5 mg/kg diet. The diet containing AFs was analyzed, and the presence of parent AFs was confirmed and determined as mentioned above.

### 2.3. Plant Material

*Calendula officinalis* was purchased from the local market at Cairo. The plant was identified by the Department of Medicinal Plants, National Research Center (NRC), and the voucher was kept in the herbarium of NRC.

### 2.4. Preparation of *Calendula* Extracts

Dried and ground flowers and leaves of *Calendula officinalis* (50 g) were subjected to extraction with 400 mL of ethanol (95%) or distilled water for 48 hrs. The extracts were filtered, and the ethanol extract was concentrated under the reduced pressure of nitrogen and completely evaporated in a vacuum oven at a temperature not exceeding 40°C until constant weights were obtained. The aqueous extract was dried using Freeze Dryer system (Dura-Dry Freeze Dryer, Model PAC-TC-V4; FTS system, Inc., Stone Ridge, NY, USA). 

### 2.5. Determination of Total Phenolic Contents

The concentration of phenolics in the extracts was determined using the method of Jayaprakasha and Rao [18]. In brief, 5 mg of each extracts was dissolved in a 10 mL mixture of acetone and water (6 : 4 v/v). Samples (0.2 mL) were mixed with 1 mL of 10-folds diluted Folin-Ciocalteu reagent and 0.8 mL of sodium carbonate solution (7.5%). The absorbance was measured at 765 nm using UV-160 IPC UV-visible spectrophotometer (Shimadzu, Japan) after 30 min at room temperature. Estimation of phenolic compounds as catechin equivalents (CE) was carried out using standard curve of catechin [19].

### 2.6. Evaluation of Radical Scavenging Activity (RSA) by 1,1-Diphenyl 1-2-Picryl Hydrazyl (DPPH) Assay

Crude extracts were dissolved in methanol to obtain a concentration of 200 ppm, and 0.2 mL of this solution was completed to 4 mL by MeOH, then 1 mL of DPPH (6.09 × 10^−5^ mol/L) solution in the same solvent was then added. The absorption was monitored after 10 min at 516 nm. The reference sample (Blank) was 1 mL of DPPH solution and 4 mL MeOH. The capacity of antioxidants to quench DPPH radical was determined according to Nogala-Kalucka et al. [20] and calculated according to the following equation:

(1) RSA  %=(Absorbance  of  control  sample     − absorbance  of  extract  sample)×(absorbance  of  control  sample)−1×100.

### 2.7. Experimental Animals

 Three-month-old male Sprague Dawley rats (100–150 g) were purchased from Animal House Colony, National Research Centre Dokki, Cairo, Egypt. Animals were maintained on standard lab diet (protein: 160.4; fat: 36.3; fiber: 41 g/kg of metabolizable energy 12.08 MJ), housed in filter-top polycarbonate cages in a room free from any source of chemical contamination, artificially illuminated (12 h dark/light cycle), and thermally controlled (25 ± 1°C) at the Animal House Lab., National Research Centre. After an acclimatization period of 1 week, the animals were divided into six groups (10 rats/group) and housed in filter-top polycarbonate cages. All animals received humane care in compliance with the guidelines of the Animal Care and Use Committee of the National Research Center.

### 2.8. Experimental Design

Animals within different treatment groups were treated daily for 6 weeks and included the control, the group fed AFs-contaminated diet (2.5 mg/kg diet), the groups treated orally with the low (CE1) and high (CE2) doses of *Calendula* extract (500 and 1000 mg/kg b.w), and the groups pretreated orally with *Calendula* extract at the two tested doses one week before and during AFs treatment for another five weeks. At the end of the treatment period, all animals were fasted for 12 hr, and blood samples were collected from the retro-orbital venous plexus from each animal under ether anesthesia. Blood samples were left to clot, and the sera were separated using cooling centrifugation at 3000 rpm for 15 min and stored at −20°C until analysis. The sera were used for the determination of ALT, AST, ALP, total protein, albumin, urea, creatinine, AFP, TNF-*α*, and IL-1*β* according to the kits instructions. 

After the collection of blood samples, all animals were killed by cervical dislocation, and sample of the liver was weighed (approximately 0.05–0.1 g) and homogenized in phosphate buffer (pH 7.4) to give 20% w/v homogenate. This homogenate was centrifuged at 1700 rpm at 4°C for 10 min, and the supernatant was stored at −70°C until analysis. This supernatant was used for the assessment of GPx, MDA, and SOD according to the kits instructions. Another sample of each liver was removed and placed in 10% of natural formalin for histological and histochemical examinations [21].

### 2.9. Statistical Analysis

All data were statistically analyzed by analysis of variance (ANOVA) using the General Linear Model Procedure of the Statistical Analysis System [22]. The significance of the differences among treatment groups was determined by Waller-Duncan k-ratio [23]. All statements of significance were based on probability of *P* ≤ 0.05.

## 3. Results 

### 3.1. Total Phenolic Content and DPPH Scavenging Activity

The results of total phenolic contents and DPPH scavenging activity of the aqueous and ethanolic extracts of *Calendula* extract are presented in Table 1. The results revealed that the total phenolic content of the ethanolic extract of *Calendula* was higher than that in the aqueous extract. Moreover, the DPPH scavenging activity of the ethanolic extract showed higher radical scavenging activity than the aqueous extract. Therefore, the ethanolic extract was used for the biological assay.

### 3.2. The Biological Assay

**Figure 1:**
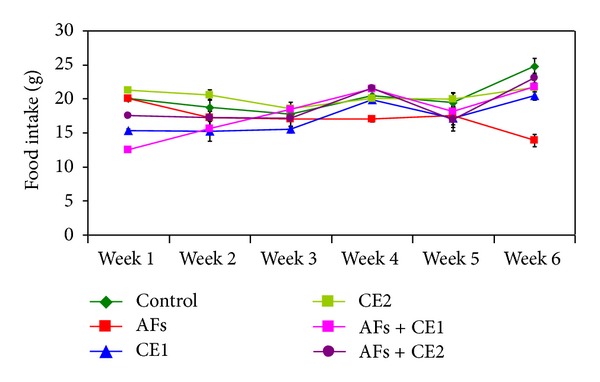
Effect of the treatment with ethanol extract of *Calendula* on food intake of rats fed AFs-contaminated diet during the experimental period (AFs: aflatoxins; CE1: low dose of *Calendula* extract (500 mg/kg b.w); CE2: high dose of *Calendula* extract (1000 mg/kg b.w)).

**Figure 2:**
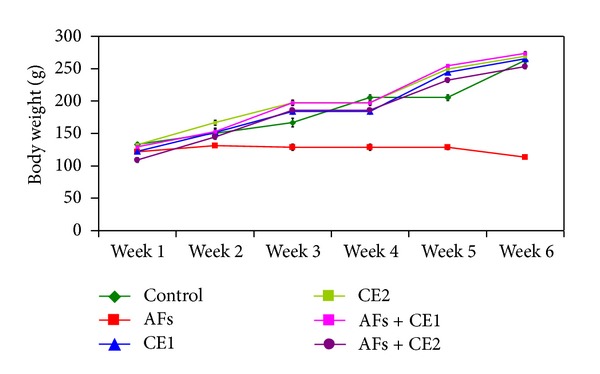
Effect of the treatment with ethanol extract of *Calendula* on body weight of rats fed AFs-contaminated diet during the experimental period (AFs: aflatoxins; CE1: low dose of *Calendula* extract (500 mg/kg b.w); CE2: high dose of *Calendula* extract (1000 mg/kg b.w)).

Along the study period, no mortality was observed in the groups treated with *Calendula* at the two tested doses either alone or one week before and during feeding AFs-contaminated diet. Effect of different treatments on the body weight changes is illustrated at Figure 1. Animals fed AFs-contaminated diet showed a significant reduction of the body weight, whereas animals treated with CE1 or CE2 were comparable to the control. CE1 or CE2 succeeded to normalize body weight when treated with animals fed AFs-contaminated diet. The results indicate that animals fed AFs-contaminated diet demonstrated a significant decrease of feed intake, but animals treated with either CE1 or CE2 were comparable to control. Groups fed AFs-contaminated diet and treated with CE1 or CE2 showed a significant improvement of feed intake. This result was pronounced in the group treated with the high dose (Figure 2).

The effects of different treatments on serum biochemical parameters are depicted in Table 2. These results indicated that animals fed AFs-contaminated diet showed a significant increase in serum ALT, AST, ALP, urea, creatinine, and AFP accompanied with a significant decrease in total protein and albumin. Animals treated with the CE1 or CE2 were comparable to the controls in all biochemical parameters. Treatment with the extract of the two tested dose one week and during feeding AFs-contaminated diet succeeded to induce a significant improvement of all biochemical parameters towards the control values. 

The existing results proved that AFs-contaminated diet resulted in a significant decrease in GPx and SOD activities accompanied with a significant increase of MDA level (Table 3). However, no significant effect was observed between those treated with CE1 or CE2 alone and the control group. Furthermore, animals fed AFs-contaminated diet and treated with CE1 or CE2 demonstrated a significant increase of GPx and SOD activities with a significant decrease of MDA level compared to the AFs-treated group. The pronounced improvement has been showed at the high dose of extract (CA2) especially in SOD which was comparable to control.

The effects of different treatments on inflammatory cytokine, TNF-*α*, and IL-1*β* were illustrated in (Table 4). AFs-treated rats showed a significant increase of the inflammatory cytokines; however, animals treated with either CE1 or CE2 were comparable to the controls. Treatment with CE1 or CE2 one week before and during AFs treatment succeeded to induce a significant improvement in TNF-*α* and IL-1*β* in a dose-dependent manner. The higher dose demonstrated a significant improvement of IL-1*β* toward the control level. 

**Figure 3:**
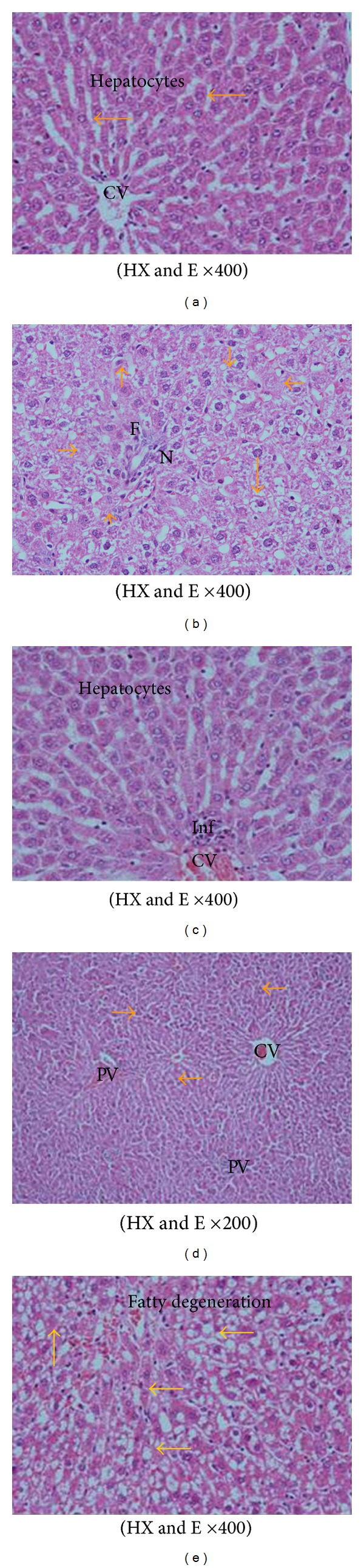
A photomicrograph in liver section from (a) a control rat branching and anatomizing cords radiating from the central vein CV. The hepatocytes that are having vesicular nuclei and some binucleated cells are separated by sinusoids lined by flat endothelial cell and kupfer cells, (b) a rat fed AFs-contaminated diet showing hepatocytes necrosis (N) and apoptosis (yellow arrows), and fibrosis around the blood vessels (F). (c) A rat treated; with CE1 or CE2 showing the normal hepatocytes with vesicular nuclei and evident mononuclear cellular infiltration around the central vein (d) a rat fed AFs-contaminated diet and treated with CE1 showing marked improvement in hepatocytes architecture; no vacuoles or fatty droplets were detected. Some cells have eosinophilic cytoplasm and deeply stained nuclei around the central and portal vein areas (arrows), and mononuclear cellular infiltration are scattered around the portal tracts. (e) A rat fed AFs-contaminated diet and treated with CE2 showing normal histological picture with few fatty degeneration and interstitial hemorrhage around the central vein.

The biochemical results obtained in the current study were confirmed by the histopathological examination of the liver tissue. Microscopic examination of the liver section of a control rat showed branching and anatomizing cords radiating from the central vein with vesicular nuclei and some binucleated cells and separated by sinusoids which lined by flat endothelial cell and kupfer cells (Figure 3(a)). The liver section of a rat fed AFs-contaminated diet showed the disorganization and damage in hepatocytes architecture accompanied with hepatocytes necrosis, apoptosis, and fibrosis around the blood vessels (Figure 3(b)). The liver section of a rat treated with CE1 or CE2 showed hepatocytes with normal architecture in the central and the portal veins accompanied with few aggregations of inflammatory cells in-between the hepatocytes (Figure 3(c)). 

 The liver section of rats treated with CE1 one week before and during AFs-treatment showed marked improvement in hepatocytes architecture; no vacuoles or fatty droplets were detected. Some cells have eosinophilic cytoplasm; and deeply stained nuclei around the central and portal vein areas and mononuclear cellular infiltration are scattered around the portal tracts (Figure 3(d)). Moreover, the liver section of rats fed AFs-contaminated diet and treated with CE2 showed normal histological picture with few fatty degeneration and interstitial hemorrhage around the central vein (Figure 3(e)).

**Figure 4:**
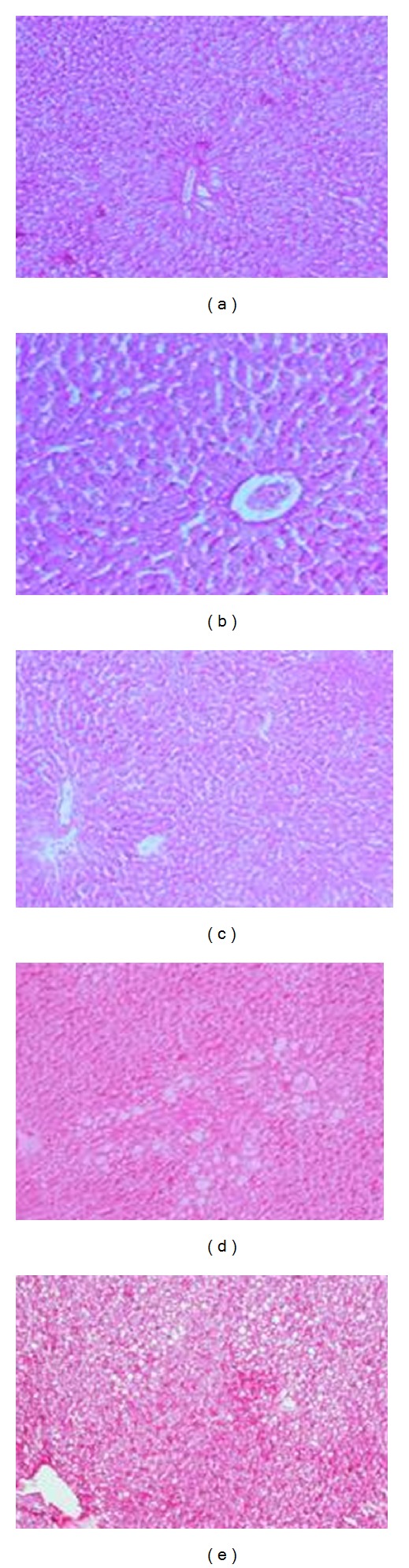
Photomicrographs in liver sections stained with Periodic-Sheif reagent-stain (PAS) for glycogen demonstration from (a) control rats showing a strong reaction of PAS, (b) rats fed AFs-contaminated diet showing a weak reaction in the damaged cell while a strong reaction in intact cells, (c) rats treated with CE1 or CE2 showing different types of reaction as well as strong reaction around the central vein and weak reaction around portal vein of hepatocytes, (d) rats fed with AFs contaminated diet treated with CE1 showing that weak reaction in some damaged cells which scattered in all the section and (e) rats fed with AFs contaminated diet and treated with CE2 showing marked improvement, although low reaction in hepatocytes were still demonstrated (PAS reaction ×100).

 The microscopic examination of liver sections stained with Periodic-Sheif reagent stain (PAS) for glycogen demonstration revealed that the control liver showed a strong reaction in the liver section (Figure 4(a)). The liver of animals fed AFs-contaminated diet showed a weak reaction in the damaged cell, while a strong reaction was noticed in intact cells (Figure 4(b)). The liver of the animals treated with CE1 or CE2 showed different types of reaction, strong reaction around the central vein, and weak reaction around portal vein of hepatocytes (Figure 4(c)). The liver of the animals fed AFs-contaminated diet and treated with CE1 showed that weak reaction in some damaged cells which scattered in all the section (Figure 4(d)). However, the liver section of animals treated with CE2 showed marked improvement, although low reaction in hepatocytes was still demonstrated (Figure 4(e)).

## 4. Discussion 

Previous reports indicated that the type of extraction of active ingredient compounds from plant material depends mainly on the type of solvent used [24]. In the current study, the total phenolic compounds of the ethanol extract of *Calendula* were higher than that in the aqueous extract. However, the ethanolic extract showed a higher DPPH (1,1-diphenyl 1-2-picryl hydrazyl) free radical scavenging activity compared to the aqueous extract. Ćetković et al. [25] showed similar scavenging potential against DPPH, hydroxyl, and peroxyl radicals and suggested that all of the extracts obtained scavenged all radicals in concentration-dependant manner. Moreover, Preethi et al. [26] tested the antioxidant potential of various leaves extracts of *Calendula* against DPPH, ABTS (2,2′-azinobis(3-ethylbenzothiazoline-6-sulfonic acid), nitric oxide, and superoxide radicals and reported lower scavenging property towards DPPH, whereas they exerted higher radical scavenging effects against the other radicals tested. 

In a similar study, Danila et al. [27] screened the water and alcoholic extracts of *Calendula* grown in Romania for its antioxidant activity and reported that *Calendula* contained a high total phenol amount and scavenging activity against DPPH and ABTS radicals. Dall'Acqua et al. [28] investigated antioxidant activity of *Calendula arvensis* used in Sardinian folk medicine using DPPH radical scavenging assay and reported that the extract showed a high radical scavenging effect towards DPPH. The radical scavenging properties were attributed to the presence of flavonoid and triterpene derivatives in the extract [29].

In the *in vivo* study, the selective dose of AFs and *Calendula* was literature-based [30, 31], respectively. The results revealed a significant reduction of body weight and food intake in animals fed AFs-contaminated diet. These results were in agreement with those reported in the literature of aflatoxicosis [32–38]. The reduction in body weight in the animals fed AFs-contaminated diet alone may be due to the effects of AFs on the balance between orexigenic and anorexigenic circuits that regulate the homeostatic loop of body weight regulation, leading to cachexia [34]. Moreover, AFs exposure may lead to significant reduction of leptin [35] accompanied with high levels of cortisol, IL-6, and insulin resistance which together act to influence the feeding response, causing weight loss in patients with pancreatic cancer [36]. Osborne et al. [37] suggested that AFs ingestion affect various digestive enzymatic activities that give rise to a malabsorption syndrome, characterized by steatorrhea, hypocarotenoidemy, and to lowering of bile, pancreatic lipase, trypsin, and amylase. Furthermore, Lesson et al. [38] reported that the 8,9 epoxide metabolite of AFB_1_ may covalently bind to DNA and proteins, which then alters enzymatic processes, such as gluconeogenesis, Krebs cycle, or fatty acid synthesis.

Animals fed AFs-contaminated diet showed a significant increase of ALT, AST, ALP, urea, and createnine which indicate changes in the hepatic tissues and biliary system [39], structural damaging of liver integrity, since these enzymes are cytoplasmic in location and released into plasma as a result of cellular damage [8, 40]. It is also likely that alteration in ALP activity affects membrane permeability and the transport of metabolites. Moreover, increase in urea and createnine level observed in the current study in AFs-treated group clearly indicated the harmful and stressful effect on renal tissue [30]. Taken together, the increased level of urea and the decreased level of albumin and total protein (TP) indicate the inhibition of protein synthesis and increase of protein catabolism and/or renal dysfunction [41, 42]. These results clearly indicated that AFs had stressful effects on the hepatic and renal tissues, consistent with those reported in the literature of aflatoxicosis [43, 44]. 

Animals treated with AFs showed a significant increase in AFP which is considered specific biomarkers for liver cancer. This increase in AFP may be due to induction of expression of mRNAs of liver alpha-fetoprotein [45, 46]. On the other hand, aflatoxin B_1_-induced hepatocarcinogenesis associated with defective DNA-damage response by passing p53 activation [47] and modulation of insulin-like growth factor 2 dependent signal axis (IGF-2) [48]. Similar to these observations, Sell et al. [49] and Yang et al. [45] reported that AFB_1_ administration resulted in the elevation of serum AFP level in both duck and rats.

The current study showed that animals fed AFs-contaminated diet suffer from oxidative stress as indicated by the significant increment of lipid peroxidation (MDA) and the significant reduction of enzymatic antioxidant such as superoxide dismutase (SOD) and glutathione peroxidase (GPx). These results are in agreement with previous studies suggested that oxidative stress may be due to direct effect of AFs or by the metabolites formed and the free radicals generated during the formation of these metabolites [34, 46, 50]. Moreover, the reduction of protein synthesis in AFs-treated animals may affect certain metal ions (i.e., iron and copper), which play an important role in free radical production and liberation [10]. In normal state, metal ions are bonded to transfer proteins, such as ceruloplasmin and transferrin [51] and play a crucial role of SOD, CAT, and GPx activities, which constitute the enzymatic antioxidant defense system of the cell, displayed bidirectional alterations, either in the form of increase or decrease, depending on the particular tissue. Previous studies demonstrated that the mechanism of AFs-induced liver injury may be due to those reactive and toxic metabolites of AFs and the liver necrosis begins when the glutathione stores are almost exhausted [34, 35, 52]. 

Tumor necrosis factor-alpha (TNF-*α*) and interleukin-1 alpha (IL-1*α*) are produced by macrophages, and they play an important role in tumor conditions [35]. TNF-*α* is an essential factor in tumor promotion [53], and IL-1 polymorphisms are important mediators in the inflammatory process [54]. In the current study, ingestion of AFs-contaminated diet significantly increased TNF-*α* and IL-1*β* suggesting that AFs preferentially affects macrophage functions. In particular, it decouples the close correlations usually observed between transcriptional and translational controls of IL-1*α* and TNF-*α* production by these cells [55]. In this concern, Barton et al. [56] stated that TNF-*α* plays a causal role in the development of liver injury, and it plays a major role in modulating mycotoxin-induced hepatotoxicity. Moreover, TNF-*α* has been proven to play an important role in inflammation in mediating the proliferation and differentiation of immune cells and development of immune response [57]. Hopkins [55] and Abdel-Wahhab et al. [35] reported that directly proportional relationship has been observed between transcriptional and translational controls of IL-1 and TNF-*α* production by hepatocytes. Consequently, the current results indicated that aflatoxicosis may induce TNF-*α* and IL-1 due to activation of caspase-3, which is one of the apoptotic mediator [58], and the significant elevation of inflammatory cytokine may affect the mRNA expression of these mediators [46]. 

The current results indicated that the ethanolic extract of *Calendula* showed a potential protective effect against AFs toxicity. Animals treated with *Calendula* extract did not show any sign of toxicity. In this concern, the safety of the ethanol extract of *Calendula* was suggested, and the oral LD_50_ in rats was estimated by 4640 mg/kg b.w [59]. The results reported herein indicated that animals treated with *Calendula* extract showed insignificant increase in food intake and body weight gain. Moreover, *Calendula* extract succeeded to induce a significant improvement in the biochemical parameters and the histological and histochemical pictures. Taken together, the results of the *in vitro* study and the *in vivo* evaluation suggested that there are an antioxidant and free radical scavenging properties of this extract, and this improvement more pronounced with the high dose (CA2) [29]. In this concern, previous study suggested that *Calendula* plays an important role in hepaticcytolysis inhibition and improves liver integrity in CCl_4_ intoxicated rats [60]. In referring to previous literature, hepatoprotective activity of *Calendula* may be due to its modulatory effect on cytochrome P450, which may interfere on aflatoxin active metabolite production [61]. Moreover, *Calendula* extract showed renoprotective action against cisplatin-induced renal toxicity through its modulatory effect against myelosuppression of the renal system. 

The existing data revealed anticarcinogenicity of *Calendula* extract indicated by significant reduction of AFP, and this effect was pronounced in the animals treated with the high dose (CA2). Several reports suggested that the anticancer properties of *Calendula* may be due to its chemoprotector properties against hepatocarcinogenesis in rats beside antitumoural activity *in vivo* in the Ehrlich mouse carcinoma model due to its ability to stimulate T-lymphocytes, B-lymphocytes, and cluster of differentiation (CD4^+^) [62]. Moreover, *Calendula* plays a crucial role in prevention of DNA damaging [63]. These results were in agreement with those reported previously [26, 31, 62]. 

The results also suggested that *Calendula* extract possesses anti-inflammatory activity which may be due to its higher content of carvacol. Carvacol has been described as a promoter for liver regeneration and inhibitor of TNF-*α* and IL-6 level in rats undergoing partial hepatectomy [64]. Furthermore, Tsai et al. [65] reported that caravacl could reduce TNF-*α* and IL-1*β* levels in intoxicated rats through the inhibition of cycloxygensae (COX) enzymes activity, mRNA expression, and its protein in lipopoly saccharides-induced inflammation. 

The histopathological and histochemical changes in liver confirmed the biochemical results and revealed that animals fed AFs-contaminated diet showed severe histological changes typical to those reported in the literature [10, 39, 66]. However, animals treated with the extract in combination with AFs resulted in a significant improvement in liver tissues similar to that reported previously [61]. 

## 5. Conclusion

It can be concluded that the ethanolic and aqueous extracts of *Calendula officinalis* have a valuable quantity of total phenolic compounds and have DPPH radical scavenging activity. Moreover, the ethanolic extract showed higher phenolic content and radical scavenging property compared to the aqueous extract. The ethanolic extract exhibited hepatorenoprotective properties against aflatoxin-induced liver injury in a dose-dependent manner due to its antioxidant, free radical scavenging activities, and anti-inflammatory properties.

## Figures and Tables

**Table 1:** Yield, total phenolic compounds and DPPH radical scavenging activity of the ethanol of *Calendula*.

Parameter	Ethanolic extract
Yield (g/100 g plant)	7.99 ± 0.49
Total phenolic compounds (*μ*g/g)	136.00 ± 21.88
DPPH radical scavenging activity (%)	96.30

**Table 2:** Effect of ethanol extract of *Calendula* on serum biochemical parameters in rats fed aflatoxins-contaminated diet.

Parameter	Groups					
	Control	AFs	CE1	CE2	CE1 + AFs	CE2 + AFs
AST (U/mL)	248.32 ± 3.14^a^	330.34 ± 4.22^d^	239.4 ± 8.96^b^	240.60 ± 6.42^a^	283.0 ± 4.2^c^	257.64 ± 3.24^a^
ALT (U/mL)	75.36 ± 1.37^a^	132.51 ± 4.05^c^	73.2 ± 2.02^a^	74.12 ± 2.69^a^	109.2 ± 1.77^b^	77.52 ± 1.33^a^
ALP (U/L)	106.75 ± 6.23^a^	234.51 ± 5.32^b^	93.67 ± 3.24^a^	104.36 ± 3.33^a^	156.71 ± 3.43^c^	111.37 ± 2.61^d^
TP (mg/dL)	9.99 ± 1.41^a^	4.35 ± 0.31^b^	9.23 ± 0.46^a^	10.44 ± 1.61^a^	8.95 ± 0.27^c^	9.27 ± 0.23^c^
Albumin (mg/dL)	3.44 ± 0.20^a^	1.88 ± 0.12^b^	3.11 ± 0.07^a^	3.37 ± 0.12^a^	3.07^c^ ± 0.14	3.16 ± 0.22^a^
Urea (mg/dL)	1.51 ± 0.14^a^	3.44 ± 0.31^b^	1.72 ± 0.13^a^	1.61 ± 0.13^a^	2.34 ± 0.12^a^	1.84 ± 0.32^a^
Creatinine (mg/dL)	0.77 ± 0.21^a^	3.33 ± 0.14^b^	0.75 ± 0.13^a^	0.76 ± 0.14^a^	2.03 ± 0.02^c^	0.87 ± 0.13^d^
AFP (ng/mL)	1.52 ± 0.78^a^	4.64 ± 0.22^b^	1.53 ± 0.17^a^	1.44 ± 0.16^a^	2.77 ± 0.31^c^	1.59 ± 0.23^a^

Within each row, means superscript with different letters are significantly different at *P* ≤ 0.05.

AFs: aflatoxins.

CE1: low dose of calendula extract (500 mg/kg b.w).

CE2: high dose of calendula extract (1000 mg/kg b.w).

**Table 3:** Effect of ethanol extract of calendula on liver antioxidants and lipid peroxidation in rats fed aflatoxins-contaminated diet.

Parameter	Group					
	Control	AFs	CE1	CE2	CE1 + AFs	CE2 + AFs
SOD (U/mg liver protein)	299.37 ± 10.33^a^	172.32 ± 7.11^b^	305.49 ± 21.67^a^	310.21 ± 8.32^a^	281.57 ± 15.04^c^	285.32 ± 9.32^a^
GPx (U/mg liver protein)	33.66 ± 2.54^a^	14.61 ± 1.20^b^	54.03 ± 1.45^c^	56.49 ± 2.12^a^	35.61 ± 1.43^a^	37.43 ± 1.56^a^
MDA (nmol/mg liver protein)	37.32 ± 2.12^a^	84.31 ± 2.44^b^	22.71 ± 2.28^c^	20.31 ± 1.44^a^	45.26 ± 1.29^d^	42.32 ± 2.13^a^

Within each row, means superscript with different letters are significantly different at *P* ≤ 0.05.

AFs: aflatoxins.

CE1: low dose of calendula extract (500 mg/kg b.w).

CE2: high dose of calendula extract (1000 mg/kg b.w).

**Table 4:** Effect of ethanol extract of calendula on serum inflammatory cytokine in rats fed aflatoxins-contaminated diet.

Parameter	Group					
	Control	AFs	CE1	CE2	CE1 + AFs	CE2 + AFs
TNF-*α* (pg/mL)	58.34 ± 3.12^a^	131.12 ± 3.32^b^	58.37 ± 2.07^a^	60.72 ± 1.92^a^	86.47 ± 2.31^c^	63.24 ± 1.66^d^
IL-1*β* (pg/mL)	0.66 ± 0.07^a^	2.34 ± 0.12^b^	0.65 ± 0.04^a^	0.84 ± 0.22^a^	1.58 ± 0.04^c^	0.77 ± 0.21^a^

Within each row, means superscript with different letters are significantly different at *P* ≤ 0.05.

AFs: aflatoxins.

CE1: low dose of calendula extract (500 mg/kg b.w).

CE2: high dose of calendula extract (1000 mg/kg b.w).
